# Developmental validation of a high-resolution panel genotyping 639 Y-chromosome SNP and InDel markers and its evolutionary features in Chinese populations

**DOI:** 10.1186/s12864-023-09709-3

**Published:** 2023-10-12

**Authors:** Guang-Bin Zhao, Lei Miao, Mengge Wang, Jia-Hui Yuan, Lan-Hai Wei, Yao-Sen Feng, Jie Zhao, Ke-Lai Kang, Chi Zhang, An-Quan Ji, Guanglin He, Le Wang

**Affiliations:** 1 National Engineering Laboratory for Forensic Science, Key Laboratory of Forensic Genetics of Ministry of Public Security, Institute of Forensic Science, Ministry of Public Security, Beijing, 100038 China; 2 School of Forensic Medicine, Kunming Medical University, Kunming, 650500 China; 3 Faculty of Forensic Medicine, Zhongshan School of Medicine, Sun Yat-sen University, Guangzhou, 510275 China; 4 School of Ethnology and Anthropology, Inner Mongolia Normal University, Inner Mongolia, 010028 China; 5 Institute of Rare Diseases, West China Hospital of Sichuan University, Sichuan University, Chengdu, 610041 China

**Keywords:** Forensic genetics, Y-chromosome, Single nucleotide polymorphism, Next-generation sequencing, Haplogroup inference

## Abstract

Uniparental-inherited haploid genetic marker of Y­chromosome single nucleotide polymorphisms (Y-SNP) have the power to provide a deep understanding of the human evolutionary past, forensic pedigree, and bio-geographical ancestry information. Several international cross-continental or regional Y-panels instead of Y-whole sequencing have recently been developed to promote Y-tools in forensic practice. However, panels based on next-generation sequencing (NGS) explicitly developed for Chinese populations are insufficient to represent the Chinese Y-chromosome genetic diversity and complex population structures, especially for Chinese-predominant haplogroup O. We developed and validated a 639-plex panel including 633 Y-SNPs and 6 Y-Insertion/deletions, which covered 573 Y haplogroups on the Y-DNA haplogroup tree. In this panel, subgroups from haplogroup O accounted for 64.4% of total inferable haplogroups. We reported the sequencing metrics of 354 libraries sequenced with this panel, with the average sequencing depth among 226 individuals being 3,741×. We illuminated the high level of concordance, accuracy, reproducibility, and specificity of the 639-plex panel and found that 610 loci were genotyped with as little as 0.03 ng of genomic DNA in the sensitivity test. 94.05% of the 639 loci were detectable in male-female mixed DNA samples with a mix ratio of 1:500. Nearly all of the loci were genotyped correctly when no more than 25 ng/μL tannic acid, 20 ng/μL humic acid, or 37.5 μM hematin was added to the amplification mixture. More than 80% of genotypes were obtained from degraded DNA samples with a degradation index of 11.76. Individuals from the same pedigree shared identical genotypes in 11 male pedigrees. Finally, we presented the complex evolutionary history of 183 northern Chinese Hans and six other Chinese populations, and found multiple founding lineages that contributed to the northern Han Chinese gene pool. The 639-plex panel proved an efficient tool for Chinese paternal studies and forensic applications.

**Supplementary Information:**

The online version contains supplementary material available at 10.1186/s12864-023-09709-3.

## Background

Single nucleotide polymorphisms (SNPs) in non­combining regions of the male-specific Y-chromosome have been used to construct a stable Y-chromosome haplogroup phylogenetic tree that is widely used for population discrimination, evolutionary studies, genetic structure analysis, and bio-geographic ancestry inference [1]. A high-resolution Y-SNP panel is a powerful tool for the studies mentioned above. Previously, SNaPshot and capillary electrophoresis methods were applied to develop multiple Y-SNP panels focused on ethnolinguistically diverse populations or particular sublineages of the Y-chromosome branches [2, 3]. However, due to some technical bottlenecks including the limitation of fluorescence labels, these methods were hardly used to analyze a large number of lineage-informative markers in a single assay, which hindered the development of panels with more comprehensive makers dominant in different continental groups or terminal markers with a higher-resolution for one population-specific lineage.

Next-generation sequencing (NGS) characterized by high­throughput is a promising methodology for detecting multiplex Y-SNPs [4–6]. Several commercial kits and in-house panels have been reported. Liu et al. studied three ethnic minorities in China with the precision ID identity panel, which contained 34 Y-SNPs assigned to major haplogroups in Y phylogenetic tree and 90 autosomal SNPs [7]. The commercial kit comprised a few Y-SNPs but was not designed for Y-chromosome study. Ralf et al. extensively validated an 859-plex Y-SNP panel using the Ion Torrent platform [6]. Claerhout et al. developed a CSYseq panel using the Illumina platform containing 1,5611 Y-SNPs [8]. However, both panels had < 5% Y-SNPs from haplogroup O, which was the dominant haplogroup in the Chinese population. These Y-SNP panels were suitable for worldwide population studies. In recent years, several panels based on the Chinese­specific tree were reported. Wang et al. developed a 165-plex Y-SNP in-house panel covering major haplogroups in Chinese populations, and the majority of Y-SNPs were used to infer haplogroups O and R [9]. To improve the resolution of the Y-SNP system, Liu et al. constructed a 265-plex customized Y-SNP panel, from which more haplogroups were inferable, including 41, 21, 31, 81, and 30 subgroups in haplogroups C, D, N, O, and R, respectively [10]. Tao et al. also developed and updated their SifaMPS 381 Y-SNP panel, including O, C, N, and D haplogroups [11]. However, all previous panels focused on worldwide populations or Chinese populations possessed the limitations of the terminal lineage coverage or the resolution of the terminal lineage. Human genomic studies based on genome-wide SNPs or high-depth whole-genome sequencing data have found that fine-scale genetic structures in China correlated with their language and geographical affiliation [12–15]. Similarly, paternal genetic structures of ethnolinguistically diverse Chinese populations were also associated with geography and language divisions. Mongolic or Tungusic speakers in the Mongolian Plateau possessed dominant lineages from Q, C, and R lineages. Speakers from the southern Chinese indigenous regions of Hmong-Mien, Tai-Kadai, Austronesian, and Austroasiatic owned complex Y-chromosome lineages derived from O1 and O2 lineages [16, 17]. Han Chinese people with the largest sample size widely distributed in China and other world regions possessed complex Y-chromosome gene pools with the O dominant lineages. Y-SNPs assigned to haplogroup O in the previously developed panels were still insufficient for lineage coverage and resolution; more O/D/C/R/Q-derived Y-SNPs should be detected for the Chinese population Y-chromosome-related lineage study.

Additionally, owing to its high sequencing throughput and accuracy, the MGI sequencing platform has gradually been applied to forensic studies [18]. Therefore, developing a Y-SNP panel with a larger number of lineage markers with higher coverage and resolution of the terminal Y-chromosome paternal lineages on the MGI platform is necessary. To develop one Y-SNP panel powerful for molecular anthropology and population genetic research, we developed and validated a 639-plex panel on the MGISEQ-2000RS platform, which contained 633 Y-SNPs and 6 Y-Insertions/deletions (Indels). We successfully covered a total of 573 Y haplogroups on the Y-DNA haplogroup tree. Most inferable haplogroups (64.4%) were subgroups of haplogroup O.

## Results

### The 639-plex panel

**Fig. 1:**
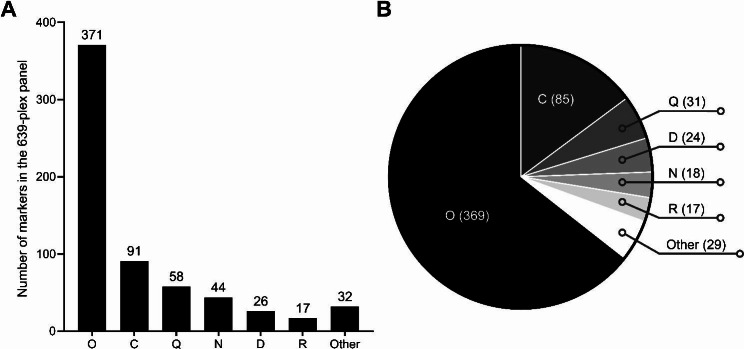
Haplogroup distribution of the 639-plex panel. (**A**) The number of markers assigned to different haplogroups in the 639-plex panel. (**B**) The number of inferable haplogroups in the 639-plex panel

The 639-plex panel genotypes 633 Y-SNPs and 6 Y-Indels, covering 573 Y-chromosome haplogroups. Y-SNP and Y-Indel loci, primer sequences, and haplogroups of the panel are given in Table S1. The panel amplifies 609 different DNA fragments in a single tube. Thirty of these amplicons were designed to genotype two of the selected Y-SNPs. The amplicon sizes ranged from 120 to 273 bp, with an average of 200 ± 12 bp (Fig. S1). Markers assigned to C, D, N, O, Q, and R haplogroups accounted for 94.99% (Fig. 1A). A total of 371 markers were haplogroup O-derived markers allowing the inference of 369 subgroups (Fig. 1B).

### MGISEQ-2000RS and MiSeq FGx sequencing metrics

A total of 354 libraries were sequenced on the MGISEQ-2000RS platform, including four libraries for concordance studies, seven libraries for accuracy and repeatability studies, 33 libraries for sensitivity studies, six libraries for specificity studies, 63 libraries for PCR inhibition studies, 15 libraries for simulated degradation studies, 41 libraries for male pedigree studies, and 185 libraries for unrelated individuals. The sequencing metrics of the four lanes were summarized in Table 1. Among the 41 male pedigree and 185 unrelated individual samples, the average depth of coverage (DOC) was 3,741×. The lowest DOC was observed in the locus F1894, which was 442×, while the highest 13,750× was observed in the locus F15400 (Fig. S2).

For comparison, one sequencing run with four libraries prepared with the 639-plex panel was conducted on a Miseq FGx machine. Run metrics are shown in Table 1. The average DOC was 315×. The lowest DOC was observed in the locus F5088, which was 27×. The highest DOC was observed in the locus SK1740, which was 1,801×. The average DOC of the loci F0588, M1843, F14184, F1759, M1842, and M479 was between the analysis threshold (54×) and detection threshold (18×).

**Table 1:** Metrics for the MGISEQ-2000RS and Miseq FGx sequencing runs in this study

Parameters	MGISEQ-2000RS					Miseq FGx
	Lane 1	Lane 2	Lane3	Lane4	Average	
Chip Productivity (%)	80.10	79.88	82.29	80.35	80.65 ± 1.11	–
Total Reads (M)	469.81	468.51	472.19	465.14	468.91 ± 2.94	14.52
Q30 (%)	84.38	84.39	78.87	84.4	83.01 ± 2.76	Read1: 94.78/Read4: 93.66
Split Rate (%)	96.43	96.41	94.34	95.17	95.58 ± 1.02	–
Lag/Phasing (%)	0.06	0.05	0.06	0.06	0.06 ± 0.01	Read1: 6.80/Read4: 5.30
Runon/Prephasing (%)	0.03	0.03	0.04	0.04	0.04 ± 0.01	Read1: 0.20/Read4: 0.00
Cluster Densities (K/mm^2^)	–	–	–	–	–	755 ± 13
Effective Spot Rate/ Cluster Passing Filter (%)	80.10	80.07	82.29	80.35	80.70 ± 1.07	92.23 ± 1.82

### Genotyping concordance between MGISEQ-2000RS and MiSeq FGx sequencing platforms

To test the genotyping concordance of the 639-plex panel on different sequencing platforms, we sequenced four genomic DNA on both MGISEQ-2000RS and Miseq FGx machines. All detectable genotypes were identical (Table S2). The loci CTS3857 and A22938 from component B of 2391c dropped out on both platforms. The locus M1732 from component C of 2391c dropped out on both platforms. The locus Z2124 dropped out in both B and C components of 2391c data on the Miseq FGx platform. Despite the presence of some drop-out loci, the inferred terminal haplogroups were consistent between different sequencing platforms.

### Accuracy and repeatability

To examine the genotyping accuracy of the 639-plex panel, we used whole genome sequencing data from four individuals for comparison. Y-SNP and Y-Indel genotypes extracted from the whole genome sequencing data proved to be consistent with the sequencing results from the 639-plex panel (Table S3). The repeatability was evaluated by sequencing three replicates of sample_B libraries with different barcodes. The results showed all three replicates obtained completely consistent genotypes (Table S4).

### Sensitivity

**Fig. 2:**
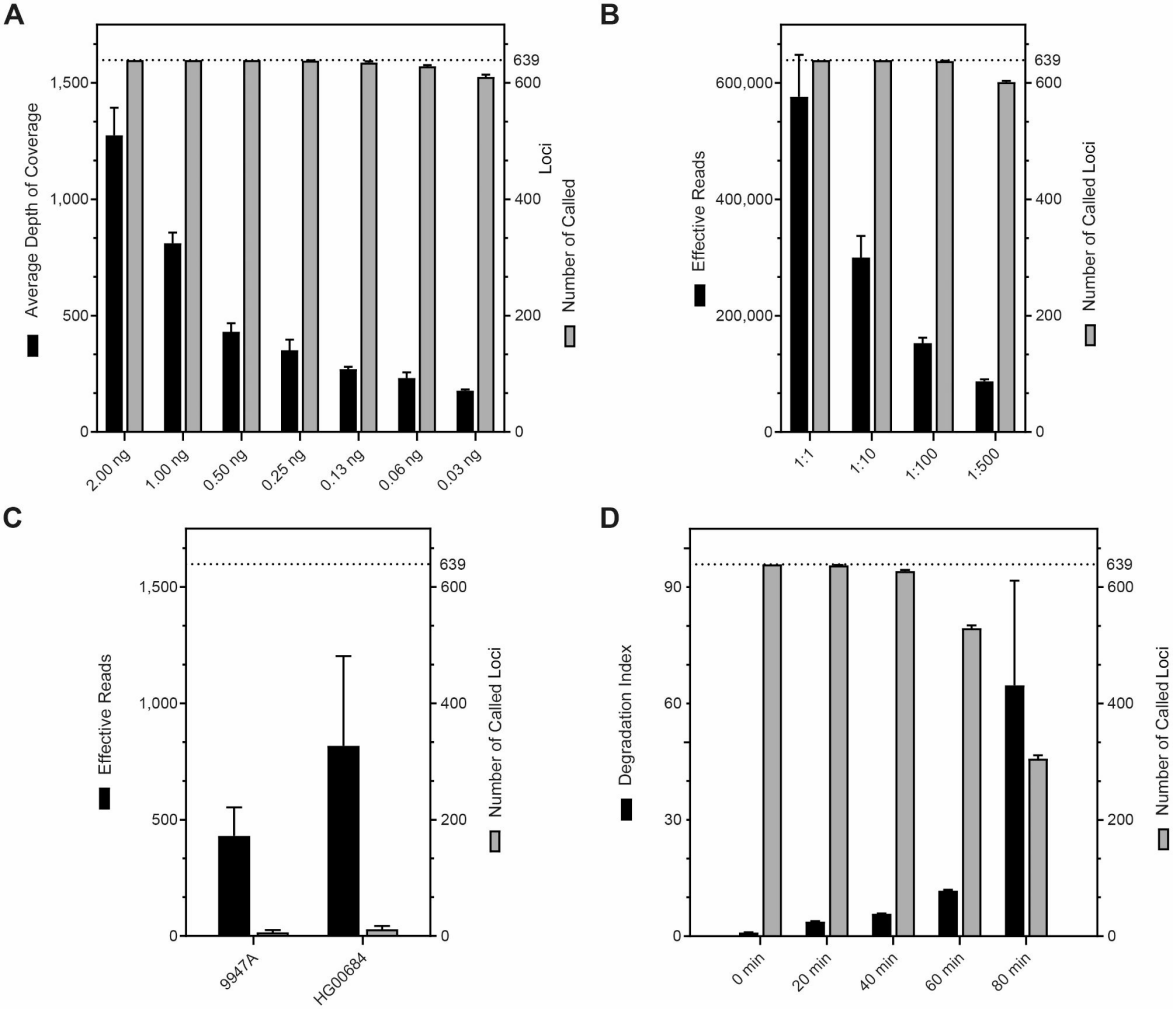
Evaluation of the 639-plex panel by sensitivity, simulated degradation, and specificity studies. (**A**) Sensitivity data using series dilutions of 2800M genomic DNA. (**B**) Sensitivity data in male-female mixed samples. (**C**) Y-chromosome specificity data using commercial female genomic DNA samples. (**D**) Simulated degradation data of the 639-plex panel

**Fig. 3:**
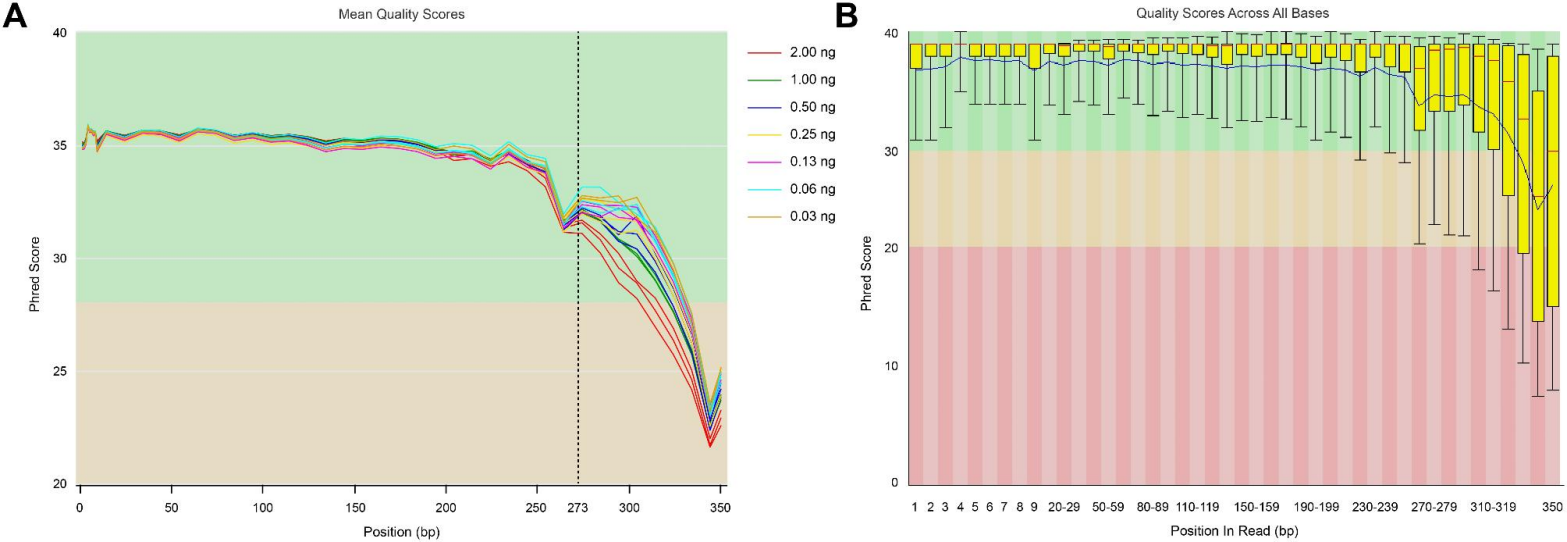
Quality scores for data in sensitivity studies. (**A**) Mean quality scores for the sequencing reads in sensitivity experiments. (**B**) FastQC profile for the sequencing reads with 0.03 ng of 2800M input DNA.

Serial dilutions of 2800M were prepared to determine the optimal amount of input DNA by evaluating the number of called loci and sequencing depth. The results revealed the average sequencing depth decreased significantly with decreasing amounts of input DNA (Fig. 2A). With as little as 0.03 ng of input DNA, 610 ± 4 loci (the mean ± standard deviation) were called. FastQC and MultiQC were used to check the quality of the sensitivity data. The mean quality scores of the reads for all samples decreased gradually with the extension of sequencing reads but remained above Q30 (Fig. 3A), indicating the base-calling accuracy was above 99.9%. There were no significant differences in the mean quality score among the samples with different amounts of input DNA. The quality scores across all bases in one library with 0.03 ng of input DNA were presented in Fig. 3B. The panel obtained reliable sequencing quality when detecting at least 0.03 ng of DNA.

DNA mixtures containing 1 ng of 2800M and four different amounts of female DNA (1, 10, 100, and 500 ng) were sequenced to assess the panel’s sensitivity under extreme male-female mixed ratios. The results showed the effective reads had decreased significantly with increasing female DNA input. However, there was no significant effect on the locus detection rate of 2800M. With the presence of 1 ng, 10 ng, 100 ng, and 500 ng of female DNA in the mixture, the detection rates of 639 loci for 2800M were 100%, 100%, 99.8%, and 94.05%, respectively (Fig. 2B).

### Y-chromosome specificity

Two female genomic DNA samples were employed to confirm the specificity of the 639-plex panel for the Y-chromosome. The total effective reads for samples 9947A and HG00684 were 430× ± 256× and 818× ± 385×, calling 6 ± 4 and 12 ± 6 loci, respectively. Effective reads of the two female samples were much lower than the results of 1ng of 2800M (533,894× ± 24,056×). Thirteen loci (F1635, F1658, MF1022, F3916, Z25928, SK1573, SK1740, F789, MF8794, F15400, M1793, CTS1350, and F1370) were genotyped two to six times in the six libraries of the two female samples, and the genotypes were identical. However, the sequencing depths of the several called loci for 9947A were all below 100×, and the sequencing depths of only two loci for HG00684 were above 100× (Fig. 2C).

### PCR inhibition

**Fig. 4:**
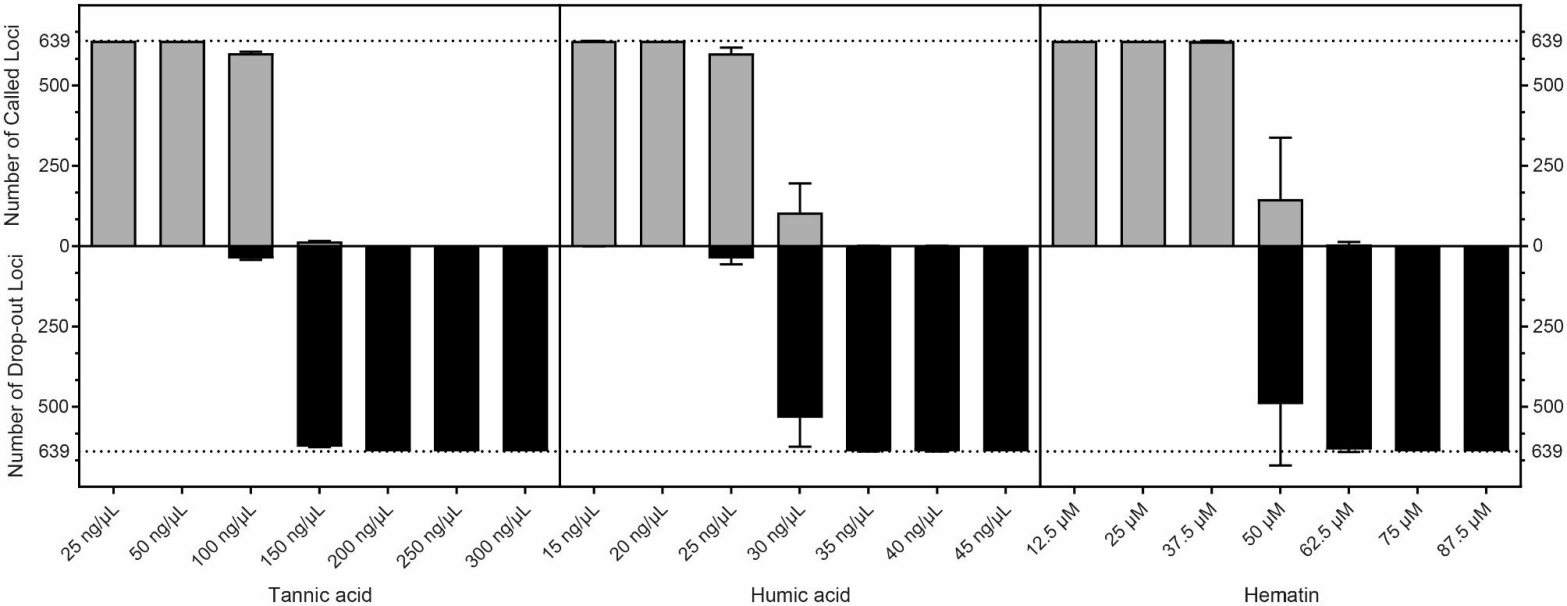
PCR inhibition studies of the 639-plex panel

Different gradients of inhibitors were added to PCR reactions to investigate the effects of three common PCR inhibitors on the amplification efficiency of the 639-plex panel. The results showed that nearly 100% of loci were detectable with no more than 50 ng/μL tannic acid, 20 ng/μL humic acid, or 37.5 μM hematin added to the amplification mixture. The mean detection rate was 93.89% when the input tannic acid concentration was 100 ng/μL, which was similar to the result of input humic acid at 25 ng/μL. Less than 25% of the loci were genotyped when tannic acid, humic acid, and hematin concentrations were over 150 ng/μL, 30 ng/μL, and 50 μM, respectively (Fig. 4).

### Simulated degradation

This assay aimed to estimate the ability of the 639-plex panel to detect degraded DNA samples. All Y-SNPs and Y-Indels were detectable when the DNA was barely degraded (DI = 0.95). With increasing fragmentation treatment time or DI values, some loci started to drop out and the average coverage depths decreased (Fig. 2D and Fig. S3). The number of called loci was 529 ± 5 when the treatment time was 60 min, and the DI value was 11.76. With the high DI value of 64.69, the number of called loci was down to 244 ± 8.

### Male pedigrees

Forty-one males from 11 pedigrees were sequenced for male pedigree studies. This work involved 79 related pairs—23 parent-offspring, 12 full siblings, 24 2nd-degree relatives, 13 3rd-degree relatives, four 4th-degree relatives, two 5th-degree relatives, and one 6th-degree relative (Fig. S4). The results showed that the detected genotypes and the inferred haplogroups from male individuals in the same pedigree were identical (Table S5). No mutation event was observed at any of the 639 loci among 11 pedigrees.

### Y-chromosome marker genotyping and haplogroup classification of unrelated Chinese Han individuals

**Fig. 5:**
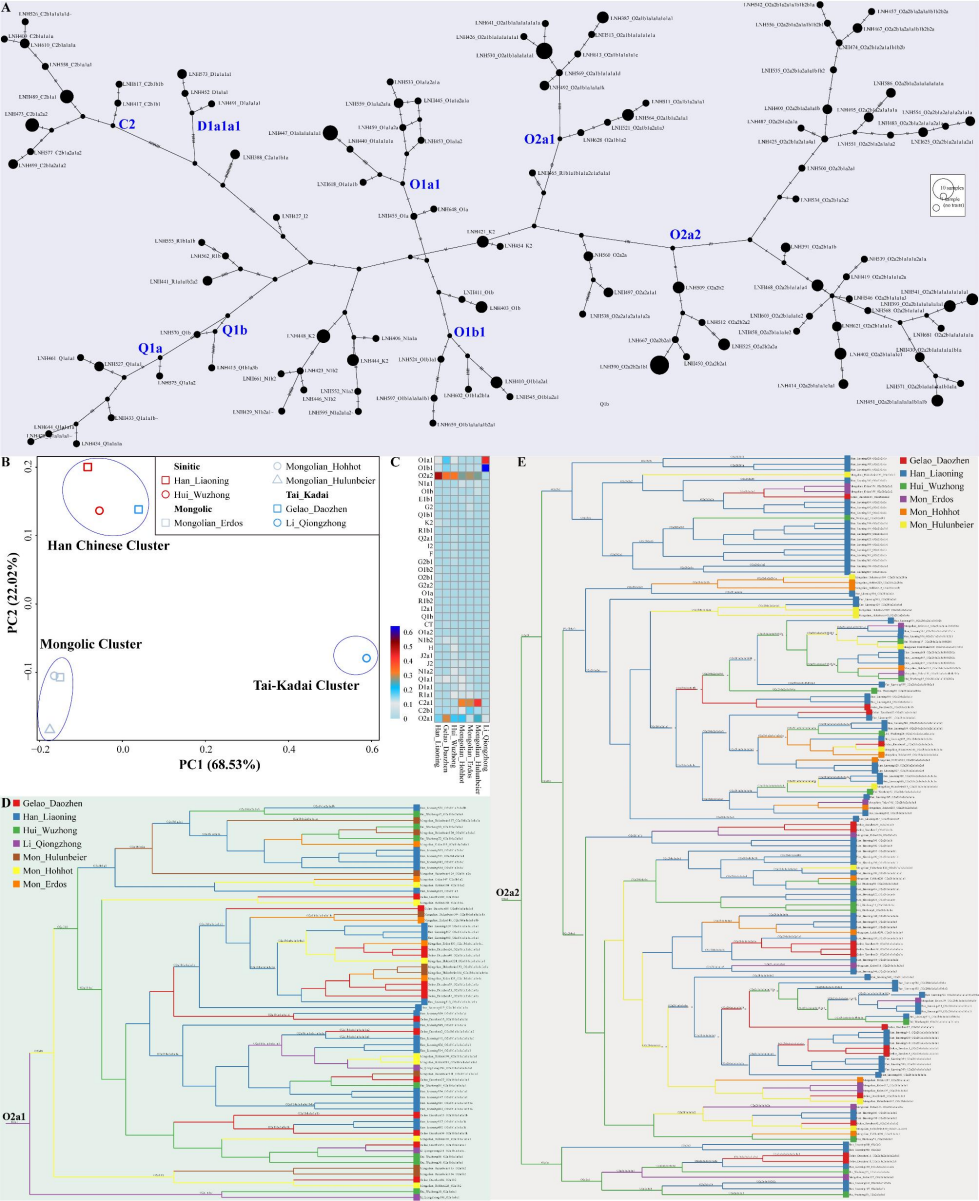
Fine-scale genetic history of Chinese populations inferred from our developed Y-chromosome panel. (**A**) Network relationship of major Y-chromosome lineages observed in Liaoning Han populations. (**B**) Principal component analysis results among seven Chinese populations. (**C**) The heatmap showed the allele frequency spectrum of Chinese people. (**D** ~ **E**) Phylogenetic relationship reconstructed based on the sequence variations

**Fig. 6:**
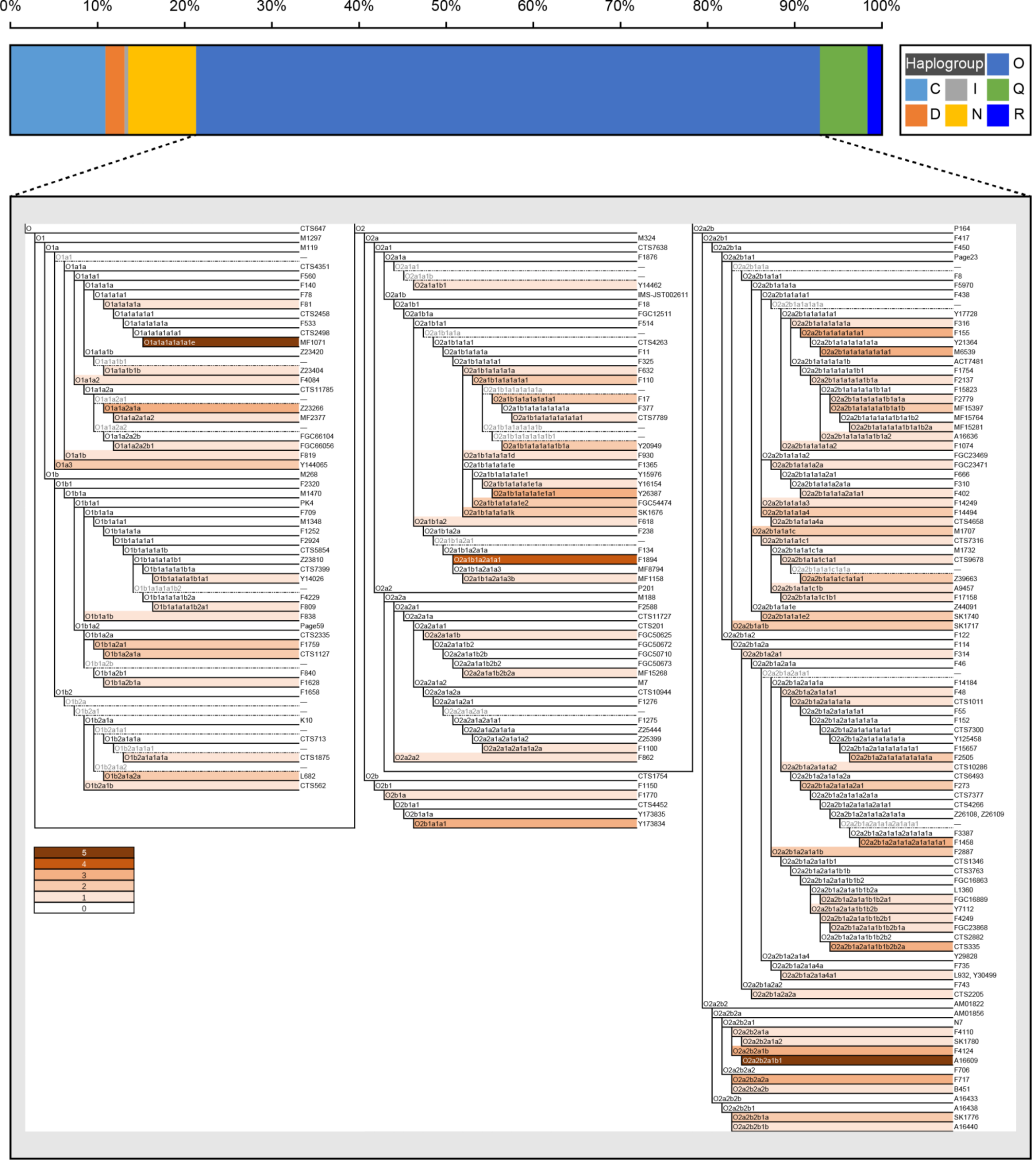
Haplogroup distribution and all observed derived markers associated with haplogroup O among the 183 unrelated Liaoning Han individuals. Five gradients of orange blocks represented the frequency of detected terminal haplogroups. Haplogroups above dotted lines were not covered in the panel

We genotyped 183 Liaoning Han individuals using our newly developed 639-plex panel to explore the resolution of this panel for paternal lineage classification in Chinese populations. We observed 118 distinct Y-chromosomal lineages in Liaoning Han with the haplogroup frequency ranging from 0.0055 (singleton) to 0.0273 (five times) (Fig. 5A). The haplogroup diversity was 0.9942. Seventy-six haplogroups were only observed once, mainly including sub-haplogroups belonging to C, D, I, N, O, Q, and R (Fig. 6 and Table S6). Twenty-five haplogroups (C2a1a1b1a, C2b1a1b1a, C2b1a1b1a1, N1a2a, N1b1b, O1a3, O1b1a2a1, O1b1a2a1a, O1b2a1a2a, O2a1b1a1a1a1a1, O2a1b1a1a1a1a1a1, O2a1b1a1a1a1a1b1a, O2a1b1a1a1a1e2, O2a1b1a1a1a1k, O2a2b1a1a1a1a1b1a1b, O2a2b1a1a1a4, O2a2b1a1a1c, O2a2b1a1a1c1a1a1, O2a2b1a1a1e2, O2a2b1a1b, O2a2b1a2a1a1a1a1a1a1a, O2a2b1a2a1a1a2a1, O2a2b1a2a1a1b, O2a2b2b1a, and Q1a1a1a) were observed twice. Thirteen haplogroups (C2b1a2a1a2, C2b1a2a2, N1a1b, O1a1a2a1a, O2a1b1a1a1a1e1a1, O2a2b1a1a1a1a1a1, O2a2b1a1a1a1a1a1a1, O2a2b1a2a1a1a2a1a1a1a1, O2a2b1a2a1a1b1b2b2a, O2a2b2a1b, O2a2b2a2a, O2b1a1a1, and Q1a1a1) were observed three times. Two haplogroups (N1b2 and O2a1b1a2a1a1) were observed four times. With a number of five, O1a1a1a1a1a1e and O2a2b2a1b1 were the most frequent terminal haplogroups.

The O haplogroup had the highest frequency in the detected Chinese Han individuals, accounting for 71.6%. Individuals from haplogroups C, D, I, N, Q, and R accounted for 10.9%, 2.2%, 0.5%, 7.7%, 5.5%, and 1.6%, respectively. All observed derived markers associated with the O haplogroup among 183 individuals were shown in hierarchical order (Fig. 6). We evaluated the distribution of upstream subgroups in the O haplogroup. Compared with the O1 subgroup, O2 had a higher frequency (78.6%). At the third level of the O haplogroup, O2a accounted for the greatest percentage of 75.6%.

### The paternal fine-scale genetic structure of Northeast Han Chinese revealed by high‑resolution Y‑chromosomal lineages

We merged our newly-generated data with previously published genotype data of 639 loci from Mongolic-speaking Mongolian, Sinitic-speaking Hui, Tai-Kadai-speaking Gelao, and Li populations to dissect the genetic relationships between northern Han and other reference populations [19]. We first explored the genetic affinity between Liaoning Han and East Asian reference populations based on the haplogroup frequency spectrum (HFS) at level 4. We found that the patterns of genetic clustering were broadly consistent with the language classifications, and Liaoning Han showed a strong genetic relatedness with Wuzhong Hui (Fig. 5B). Surprisingly, Daozhen Gelao showed a closer relationship with Sinitic-speaking people from North China rather than with linguistically close Qiongzhong Li. An early population genetic study suggested that Tai-Kadai-speaking Gelaos shared more alleles with ancestral northern East Asians relative to ancestral southern East Asians, while the opposite was true for Li [20], which may cause the differentiated population structure between these two Tai-Kadai-speaking populations and the close genetic relatedness between Daozhen Gelao and geographically distant Sinitic-speaking people. Mongolic-speaking populations separated from the Han Chinese cluster and Tai-Kadai-speaking Li people. The HFS at level 4 revealed similar patterns of haplogroup distribution between Liaoning Han and Wuzhong Hui (Fig. 5C), consistent with the clustering patterns disclosed by PCA. We observed that O2a1 (0.1202) and O2a2 (0.4153) were the dominant paternal lineages in Liaoning Han (Fig. 5A and C). The phylogenetic topologies showed that O2a1 also occupied a considerable proportion in Tai-Kadai-speaking Gelao and Mongolic-speaking Mongolian populations (Fig. 5D), and O2a2 also accounted for a considerable proportion in other East Asian populations (Fig. 5E), indicating that Han-related ancestry contributed substantially to the gene pools of ethno-linguistically diverse East Asian populations. Additionally, we found that Mongolian-dominant lineages of C2a1 and C2b1, Tibetan-dominant lineage of D1a1, Northeast Asian-derived lineages of N1a1 and N1a2, Tibeto-Burman-prevailing lineage of N1b1, southern East Asian/Southeast Asian-prevailing lineages of O1a and O1b, Siberian-derived lineages of Q1a and Q1b, and West Eurasian-derived lineages of I2, R1a and R1b contributed to the mosaic patterns of paternal lineages of Liaoning Han (Fig. 5A and C), suggesting extensive gene flow between Han-related ancestry and other ancestral East Asian populations [21–26].

## Discussion

The 639-plex panel with short amplicon size and high­resolution in the Y haplogroup was very suitable for forensic applications and population structure studies, particularly in the Chinese population. This panel can be used on the MGI and Illumina sequencing platforms, providing a flexible Y-SNP/Y-Indel detection strategy for NGS laboratories. In contrast to other studies [6, 8–11], more Y-SNPs derived from haplogroup O were obtained, and more subgroups from haplogroup O were inferable. When compared to the Ion AmpliSeq HID Y-SNP Research Panel v1 [6], a commercial Y-SNP panel concentrated primarily on the markers in haplogroups R (20.63%), E (12.19%), and I (9.69%), 5.5% were useable in haplogroup O. Although the CSYseq panel could be used to distinguish 1,443 haplogroups, 6.37% were useable in haplogroup O [8]. The two systems were more suitable for worldwide haplogroup inference. The 639-plex Y-SNP panel could analyze ~ 11 times as many O haplogroups as the Ion AmpliSeq HID Y­SNP Research Panel v1 and ~ 4 times as many as the CSYseq panel, which was more suitable for Chinese haplogroup inference.

In recent months, for haplogroup analysis in Chinese populations, Liu et al. [10] and Tao et al. [11] reported a 256-plex Y-SNP panel including 81 haplogroup O-derived Y-SNPs and a SifaMPS 381 Y-SNP panel including 224 haplogroup O-derived Y-SNPs, respectively. Compared to these panels, the 639-plex panel obtained higher resolution in haplogroup O. For example, three and two subgroups of haplogroup O1a1a1a1a1 were inferable in the 256-plex Y-SNP panel and the SifaMPS 381 Y-SNP panel, respectively; however, 23 subgroups were inferable in the 639-plex panel, which could be a useful tool to analyze the genetic structure in haplogroup O1a1a1a1a1 further.

To reflect the higher resolution of the 639-plex panel in haplogroup O, we chose the sample DL-416 collected in 183 unrelated individuals (Table S6) that was classified into the haplogroup O1b2a1a1a1a by the 639-plex panel and inferred its haplogroups in other panels according to the Y haplogroup trees constructed by corresponding panels. Inferable terminal haplogroups of the sample DL-416 in the Ion AmpliSeq HID Y-SNP Research Panel v1, the CSYseq panel, the 256-plex Y-SNP panel, and the SifaMPS 381 Y-SNP panel were O1b2a1a, O1b2a1a1, O1b2, and O1b2, respectively.

In the sensitivity experiments, if the amount of input DNA was reduced to 0.03 ng, the 639-plex panel could still genotype more than 95.46% of the loci. This percentage was higher than 67% reported in the Ion AmpliSeq HID Y-SNP Research Panel v1 with 0.05 ng input DNA [6], 93.0% in the 256-plex panel with 0.05 ng input DNA [10], and 51.3% in the SifaMPS 381 Y-SNP panel with 0.08 ng input DNA [11].

The Y-chromosome specificity results showed that markers in the 639-plex panel were not applicable to genotype female samples. Although a few of Y-SNPs were called, the DOC of most loci was < 100×. This situation might be caused by slight contamination.

## Conclusion

Since the first whole-genome sequences were published, human genomic studies in the past two decades have changed our understanding of the patterns of genetic diversity, such as Africans possessing the highest genetic diversity and complex Linkage-Disequlibrium pattern. However, previous human genetic studies majorly focused on European ancestry for disease risk prediction models, forensic panel development and other fine-scale anthropological research. To promote the representation of genetic diversity of Chinese populations and provide a new tool with higher resolution for forensic pedigree study, we developed and validated the 639-plex panel, including 633 Y-SNPs and 6 Y-Insertion/deletions, which possessed higher coverage and resolution of terminal Y-lineages. The estimates of our validation tests showed a highly powerful performance of the panel, suggesting that the 639-plex panel is a powerful tool for Y-chromosome­related forensic applications and haplogroup inference in the Chinese population. Whole-genome sequencing data from Chinese populations in future cohort studies would revise the final phylogenetic trees of Chinese populations, which would provide more new lineage-informative markers for the next generation of this 639-plex panel.

## Materials and methods

### Marker selection and primer design

Two sources were used to screen the most comprehensive Y-SNPs in this panel: the public and in-house databases. Firstly, initial candidate Y-SNP and Y-Indel markers were from the International Society of Genetic Genealogy (ISOGG) Y-DNA Haplogroup Tree 2019-2020 (version 15.73) (https://isogg.org/tree/index.html), 1000 Genomes Project (https://ncbi.nlm.nih.gov/variation/tools/1000genomes/), and Y Chromosome Haplotype Reference Database (YHRD) (https://yhrd.org/), and Yfull databases (https://www.yfull.com/). Secondly, we constructed the in-house database collected whole-genome sequencing data from the pilot work of 100 K genome sequencing of rare disease (100KGSRD^WCH^), 10 K Chinese Person Genomic Diversity Project (10K_CPGDP), Human Genetic Diversity Project (HGDP) [27], the expanded 1000 Genomes Project cohort [28]. We used our whole-Y-chromosome sequence to construct the fine-scale revised phylogenetic tree with recalibrated divergence times and population allele frequency for each terminal lineage, which can help to choose better Y-chromosome SNPs for panel development. Generally, Y-SNPs and Y-Indels were screened out according to the following principles: (1) the markers were polymorphic for Chinese populations; (2) the haplogroup distribution of these markers was concentrated in C, D, N, O, Q, and R haplogroups, especially the O haplogroup with a population allele frequency larger 5% and divergence time older than 500 years; (3) no reverse mutations were reported for the selected markers.

Primers were designed using the Primer Premier 5.0 software [29], and the amplicon sizes were mainly concentrated at 200 bp. The specificity of primers was verified by MFEprimer 3.0 [30]. Optimal primers, primer concentrations, and thermal cycling conditions were selected after several rounds of adjustments. A total of 633 Y-SNPs and 6 Y-Indels were amplified in a single multiplex primer pool.

### Sample preparation

Several types of commercial male and female genomic DNA samples were used in this study. Male genomic DNA products included 2800M (Promega, Madison, WI, USA), components B and C of the 2391c standard reference material® (NIST, Gaithersburg, MD, USA), M2 (NuHigh Biotechnologies, Suzhou, Jiangsu, China), HG00698 (Coriell Institute, Camden, NJ, USA), and NA18624 (Coriell Institute). Female genomic DNA samples included 9947A (Promega) and HG00684 (Coriell Institute). HG00698 and NA18624 have been sequenced in a highcoverage (30×) whole genome sequencing project [28].

Saliva samples were collected from 226 Chinese Hans (41 individuals from 11 paternal pedigrees, 183 unrelated Han Chinese living in Dalian of Liaoning Province, and two other unrelated individuals with sample names Sample_A and Sample_B). This study was approved by the Ethics Review Board of the Institute of Forensic Science, Ministry of Public Security of China, and all sample donors gave written informed consent. DNA was extracted using the PrepFiler™ express BTA forensic DNA Extraction Kit (Thermo Fisher Scientific, Waltham, MA, USA) and quantified with a Qubit® 3.0 Fluorometer (Thermo Fisher Scientific) using the Qubit® dsDNA High­Sensitivity Assay Kit (Thermo Fisher Scientific) following the manufacturer’s recommendations.

Concordance—2800M, components B and C of the 2391c standard reference material®, and M2 were detected on the MGISEQ-2000RS (MGI, Shenzhen, Guangdong, China) and Miseq FGx (Illumina, San Diego, CA, USA) platforms.

Accuracy and repeatability—Sample_A, Sample_B, HG00698, and NA18624 were genotyped for accuracy studies. Three replicates of sample_B with 1 ng of input DNA were used for repeatability studies, and all these libraries were sequenced in the same run.

Sensitivity—2800M and HG00684 were used for sensitivity assessment. Libraries were prepared in triplicate with seven different amounts of 2800M (2.00, 1.00, 0.50, 0.25, 0.13, 0.06, and 0.03 ng) and sequenced. Additionally, libraries were prepared with mixtures of 1 ng of 2800M and 1 μL of HG00684 (1, 10, 100, and 500 ng/μL) in triplicate and sequenced.

Specificity—9947A and HG00684 female genomic DNA samples were used for Y-chromosome specificity studies. Libraries were prepared in triplicate and sequenced in the same run.

Polymerase chain reaction (PCR) inhibition studies—Seven gradients of hematin (87.5, 75, 62.5, 50, 37.5, 25, and 12.5 μM), tannic acid (300, 250, 200, 150, 100, 50, and 25 ng/μL), and humic acid (45, 40, 35, 30, 25, 20, and 15 ng/μL) were prepared for PCR inhibitor studies. For each gradient inhibitor experiment, 1 μL of inhibitor and 1 ng of 2800M were added to the reaction mixture. Each gradient was prepared in three replicates in parallel and sequenced.

Simulated degradation—A total of 100 μL (1 ng/μL) 2800 M was fragmented using an XM-26A ultrasonic instrument (Xiao Mei Chao Sheng, Kunshan, Jiangsu, China). The DNA solution was sheared with a power of 1000 W for 0, 20, 40, 60, and 80 min. At each time point, 10 μL of DNA was taken out for detection. The degradation index (DI) was estimated using the Quantifiler™ Trio DNA Quantification Kit (Thermo Fisher Scientific) on a 7500 Real­Time PCR System (Thermo Fisher Scientific). A 1 μL sample of fragmented DNA was used for Y-SNP genotyping in triplicate.

### Multiplex amplification

PCRs were performed with a total reaction volume of 20 μL, which included 10 μL of Master Mix (Institute of Forensic Science, Ministry of Public Security, Beijing, China), 6 μL of Primer Mix (concentrations indicated in Table S1), 3 μL of nuclease-free water (Thermo Fisher Scientific), and 1 ng of template DNA except for sensitivity studies. The reaction mixture was kept at 95 °C for 5 min, followed by 28 cycles of denaturing at 95 °C for 30 s, annealing at 59 °C for 2 min, and extension at 72 °C for 2 min, with a final elongation step at 72 °C for 2 min. The PCR products were purified with the MinElute® PCR Purification Kit (Qiagen, Hilden, Germany).

### Library preparation and sequencing

Except for the four samples (2800M, components B and C of the 2391c standard reference material®, and M2) detected by both platforms in concordant studies, all other samples were sequenced on the MGISEQ-2000RS platform.

For MGISEQ-2000RS sequencing, libraries were prepared using the MGIEasy Amplicon Library Preparation Kit (MGI) as described in a previous publication [31], and sequenced using an MGISEQ-2000RS High-throughput Sequencing Kit (MGI) with a read length set at 350 bases. For Miseq FGx sequencing, libraries were prepared using the Truseq® DNA PCR-Free HT Kit (Illumina), and quantified using the KAPA Library Quantification Kit (Roche, Basel, Switzerland) on a 7500 real­time PCR system. The MiSeq v2 Reagent Kit (300 cycles PE; Illumina) was used for sequencing.

### Sequencing data acquisition and analysis

FASTQ data was generated with the ZebraV2Seq_1.4.0.184 (MGI) and Miseq FGx™ Control Sofware (Version: 1.3.6744.33558; Verogon), respectively. The SNPTyper software [31] was used for Y-SNP allele calling and sequencing depth statistics. The detection threshold was set at 18 reads, and the analysis threshold was 54 reads. Genotypes below the detection threshold were filtered out. Genotypes with a depth of coverage between the detection and analysis threshold were manually reviewed to determine whether to be retained or not. Y-Indel alleles were manually called after visualization with Integrative Genomics Viewer (Version: 2.8.10) [32]. Reads were aligned to the human reference genome GRCh38. FastQC software [33] was applied for data quality control, and MultiQC software [34] was used to compare data quality between different samples. Genotypes were compared to the Y phylogenetic tree for haplogroup assignment manually.

A Variant Call Format (VCF) file, including all variants in the HG00698 and NA18624 genome, was downloaded from the International Genome Sample Resource (ftp://ftp.1000genomes.ebi.ac.uk/vol1/ftp/data_collections/1000G_2504_high_coverage/working/20201028_3202_raw_GT_with_annot/20201028_CCDG_14151_B01_GRM_WGS_2020-08-05_chrY.recalibrated_variants.vcf.gz). Genotypes of 633 Y-SNPs and 6 Y-Indels were extracted from the VCF file for comparison with the data sequenced with the 639-plex panel. The Microsoft Excel software (version 2308, Microsoft Corp., Redmond, WA, USA) was used for data comparison.

For population genetic analysis, previously published 334 Y-chromosome variation data from Hui, Gelao, Li, and three Mongolian populations was employed [19]. We estimated the haplogroup allele frequency in different level of terminal haplogroups and calculated the Fst genetic distances based on the allele frequency. We used principal component analysis and heatmap to explore the genetic relationship between Liaoning Han and other reference Chinese populations. We used popART [35] to explore the phylogenetic relationship of different ethnic populations based on the shared haplotypes or haplogroups and reconstructed the phylogenetic topology using Y-LineageTracker [36]. Haplogroup diversity (H) was estimated by *H = N(1-∑x*_*i*_^*2*^*)/(N-1)*, where *x*_*i*_ represents the haplogroup frequency, and *N* represents the sample size [37].

### Whole genome sequencing and variant calling

Whole genome sequencing of Sample_A and Sample_B was performed at Annoroad Gene Technology (Beijing, China) using the DNBSEQ-T7 (MGI) platform. The target depth was 100× per sample. FASTQ data were aligned with the BWA-MEM tool [38]. Obtained SAM files were converted to BAM files and sorted by the SAMtools software [39]. Variants were called according to the GATK best practices pipeline [40]. A VCF file containing all variants was obtained for data comparison.

### Electronic supplementary material

Below is the link to the electronic supplementary material.

Supplementary Material 1

Supplementary Material 2

Supplementary Material 3

Supplementary Material 4

Supplementary Material 5

Supplementary Material 6

Supplementary Material 7

Supplementary Material 8

Supplementary Material 9

Supplementary Material 10

## Data Availability

All data generated or analyzed during this study are included in this published article and its supplementary information files.

## Abbreviations

DI: Degradation index
DOC: Depth of coverage
Indel: Insertions/deletion
NGS: Next-generation sequencing
SNP: Single nucleotide polymorphism
Y-SNP: Y-chromosome single nucleotide polymorphism
