# Supplementary figures and images for: Developmental validation of a high-resolution panel genotyping 639 Y-chromosome SNP and InDel markers and its evolutionary features in Chinese populations
Source: BMC Genomics. 2023 Oct 12;24:611. doi: 10.1186/s12864-023-09709-3 (PMC10568895; doi:10.1186/s12864-023-09709-3)

**
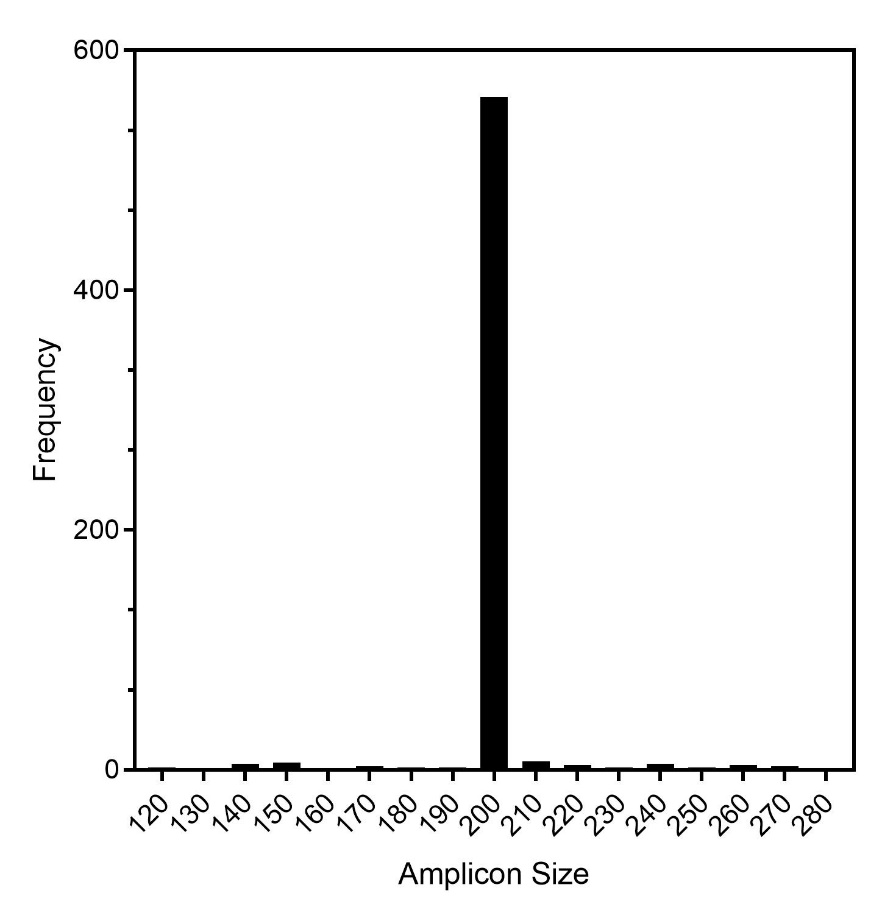
**

**Fig. S1. Amplicon size distribution of the 639-plex panel.**

Supplement: Supplementary file 1 — Supplementary Material 1 [file 12864_2023_9709_MOESM1_ESM.docx]

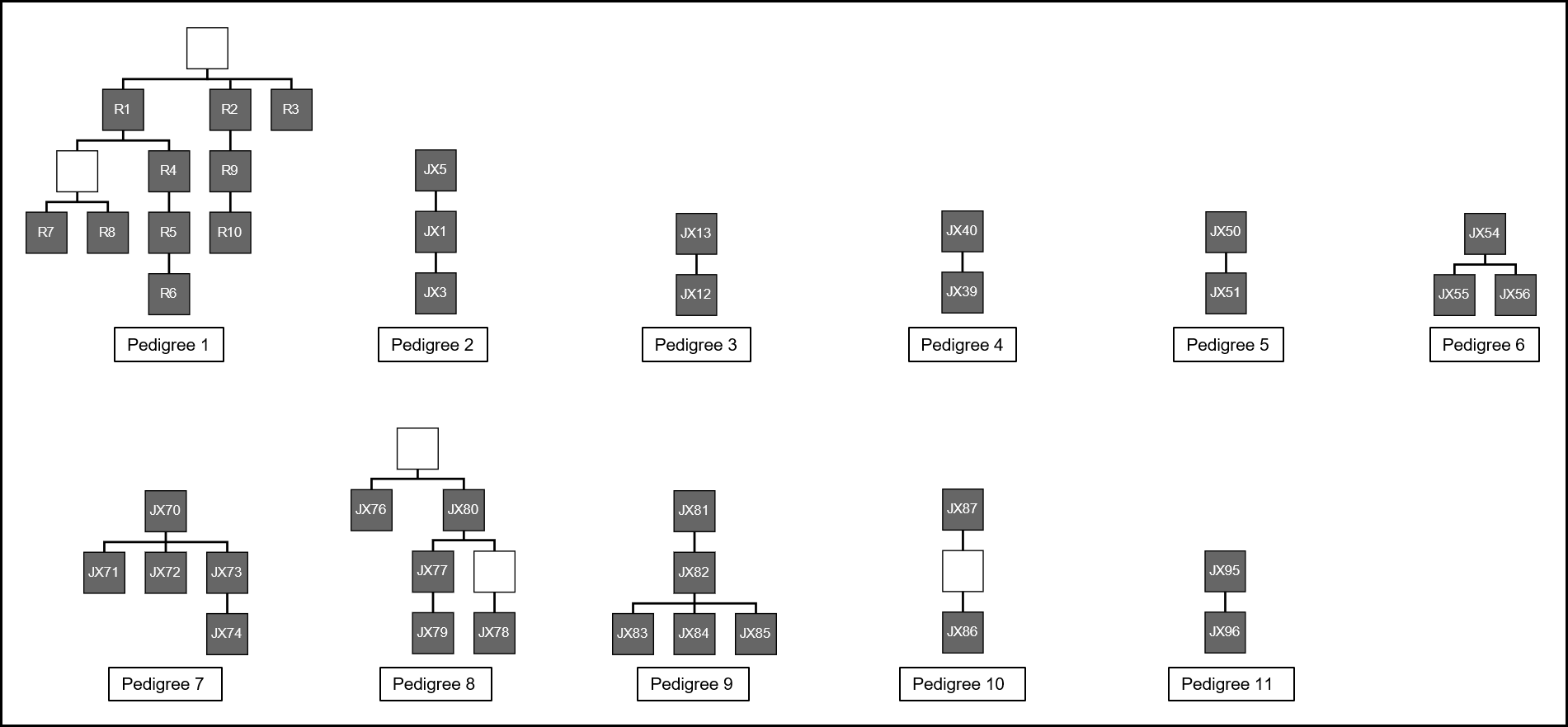


**Fig. S4. Forty-one samples in eleven pedigrees used in this study.**

Supplement: Supplementary file 4 — Supplementary Material 4 [file 12864_2023_9709_MOESM4_ESM.docx]
