# Supplementary material for: Developmental validation of a high-resolution panel genotyping 639 Y-chromosome SNP and InDel markers and its evolutionary features in Chinese populations
Source: BMC Genomics. 2023 Oct 12;24:611. doi: 10.1186/s12864-023-09709-3 (PMC10568895; doi:10.1186/s12864-023-09709-3)
Supplement: Supplementary file 2 — Supplementary Material 2 [file 12864_2023_9709_MOESM2_ESM.docx]

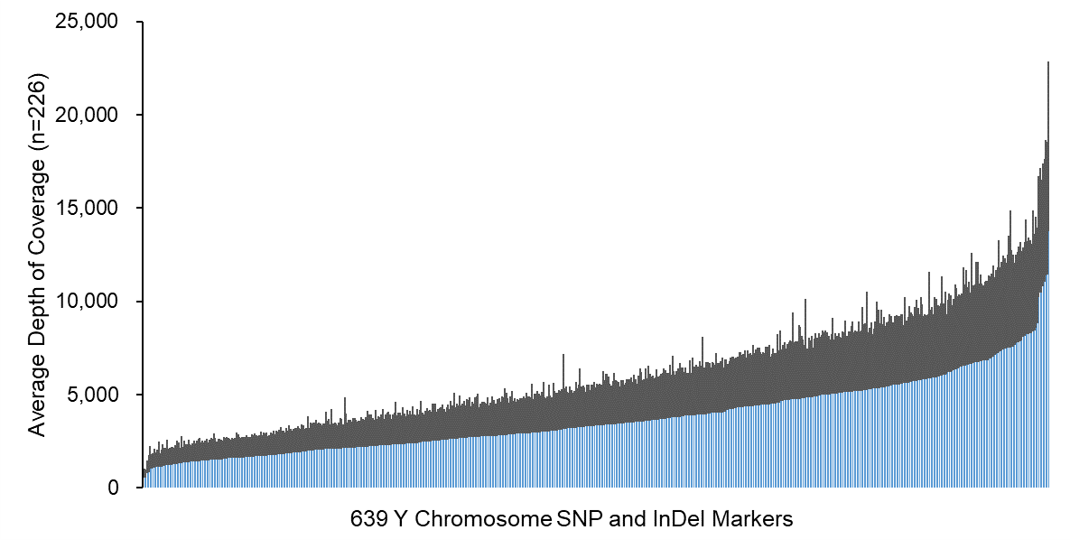


**Fig. S2. Average sequencing depth of each locus in the 639-plex panel on MGISEQ-2000RS (n=226).** The light blue and dark blue columns represented the mean depth of coverage and the standard deviation of each locus.
