# Supplementary material for: Developmental validation of a high-resolution panel genotyping 639 Y-chromosome SNP and InDel markers and its evolutionary features in Chinese populations
Source: BMC Genomics. 2023 Oct 12;24:611. doi: 10.1186/s12864-023-09709-3 (PMC10568895; doi:10.1186/s12864-023-09709-3)
Supplement: Supplementary file 3 — Supplementary Material 3 [file 12864_2023_9709_MOESM3_ESM.docx]

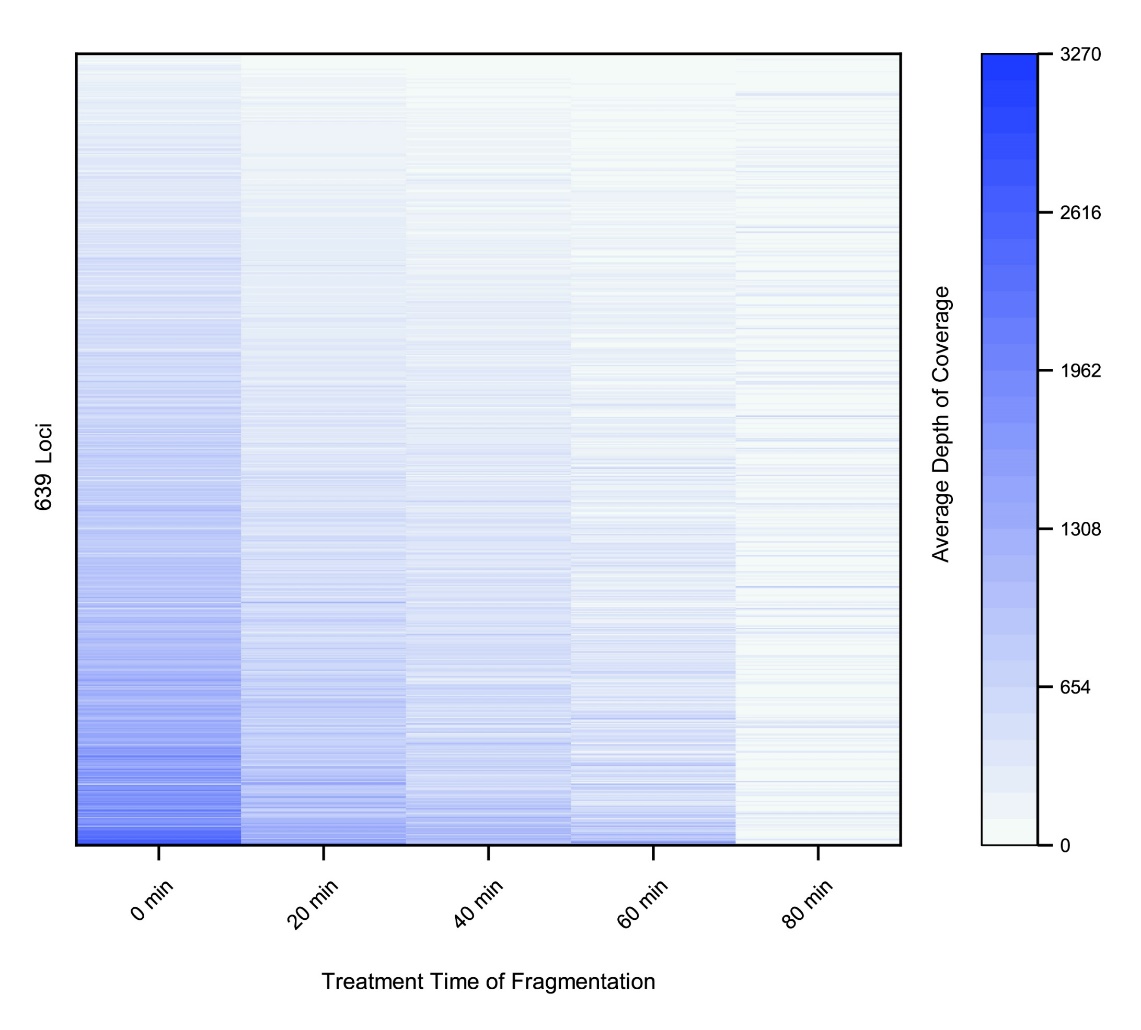


**Fig. S3. Average depth of coverage of 639 loci with different treatment time of fragmentation.** Loci were sorted according to their average depth of coverage at 0 min treatment time.
